# Colorectal cancer in Crohn’s colitis is associated with advanced tumor invasion and a poorer survival compared with ulcerative colitis: a retrospective dual-center study

**DOI:** 10.1007/s00384-020-03726-4

**Published:** 2020-09-12

**Authors:** Leonie E. Vetter, Susanne Merkel, Alan Bénard, Christian Krautz, Maximilian Brunner, Anke Mittelstädt, Nicolas Schlegel, Armin Wiegering, Christoph-Thomas Germer, Klaus Weber, Robert Grützmann, Georg F. Weber

**Affiliations:** 1 Department of Surgery, Friedrich-Alexander-University Erlangen, Krankenhausstraße 12, 91054 Erlangen, Germany; 2 Department of General, Visceral, Transplant, Vascular and Pediatric Surgery, Julius-Maximilians-University Würzburg, Oberdürrbacher Straße 6, 97080 Würzburg, Germany

**Keywords:** Crohn’s colitis, Ulcerative colitis, Colorectal cancer

## Abstract

**Purpose:**

Colorectal cancer is a well-recognized complication of inflammatory bowel diseases (IBD), such as ulcerative colitis (UC) and Crohn’s colitis (CC). In this study, we assess the clinico-pathological features and outcomes of patients with colorectal cancer from UC in comparison with CC.

**Methods:**

Data of all patients with colitis-associated cancer (CAC) who underwent surgery at Erlangen or Würzburg University Clinic between 1995 and 2015 were selected. Clinical, histopathological, and survival data were analyzed retrospectively.

**Results:**

Of all 88 patients with CAC, 20 patients had Crohn’s colitis and 68 patients had ulcerative colitis. We observed a young median age at tumor diagnosis (49.5 years UC; 45.5 years CC, *p* = 0.208) in both diseases and a long median disease duration before CAC (19 years UC; 18 years CC; *p* = 0.840). Patients with CC suffered more often from rectal cancer (14 (70.0%) in CC; 23 (33.8%) in UC; *p* = 0.005) and advanced tumor stages (8 (47.0%) pT4 in CC; 14 (25.0%) pT4/ypT4 in UC; *p* = 0.008). Five-year overall survival rate was 39.3% for CC and 67.1% for UC (*p* = 0.009 for difference between the groups). Survival did not differ significantly between UC and CC in the multivariate analysis after correction for UICC tumor stage.

**Conclusion:**

CAC in CC showed advanced tumor stages associated with reduced survival compared with CAC in UC. This may be explained by less intense surveillance in patients with CC leading to delayed cancer diagnosis.

## Introduction

Colorectal cancer is the third common malignancy worldwide with a prevalence rate of 1.4 million cases per year [1]. Chronic intestinal inflammation, as it is characteristic for the inflammatory bowel diseases (IBD) ulcerative colitis (UC) and Crohn’s colitis (CC), is a well-recognized risk factor for the development of Colorectal Cancer, called colitis-associated cancer (CAC). The likelihood of CAC increases with the disease duration as well as the extent and severity of colitis and the presence of primary sclerosing cholangitis [2]. A meta-analysis revealed a 2.4-fold overall risk of developing a colon carcinoma in UC [3] and the incidence of CAC is also more frequent in CC than among the general population with a standardized incidence ratio (SIR) of 1.9 [4, 5]. While sporadic colorectal cancer development follows a well-described adenoma-carcinoma sequence and occurs mostly in older patients, CAC affects younger patients and arises from multiple lesions in the large bowel without necessarily evolving from an adenoma [6]. Even though the pathophysiology of inflammatory carcinogenesis is not completely understood, it is well known that the mechanisms of tumor development and promotion differ from those in sporadic carcinogenesis. For example, reactive oxygen and nitrogen species (RONS), especially produced by innate immune cells in inflamed tissue, lead to early genomic alterations of the p53 tumor suppressor gene, an important protector from invasive carcinogenesis [7, 8]. Additionally, pro-inflammatory transcription factors and cytokines like NF-κB and IL-6 have been shown to play a crucial role in CAC promotion and are overexpressed in epithelial cells of patients with active colitis [9, 10]. While comparisons of sporadic colorectal cancer with CAC regarding tumor characteristics and outcome has widely been reported, only few studies exist—to the best of our knowledge—that compare colorectal cancer in patients with UC with those with CC. In one study, the authors reported a high amount of advanced cancer stages in both groups and a poorer survival for CC, although the difference did not reach a level of significance [11]. In this paper, we compare the clinico-pathological features and outcome of CAC in patients with UC with those with CC in a dual-center study using retrospective analysis.

## Methods

For this retrospective dual-center study, patients with CAC were selected from the clinical databases of the Department of Surgery at Erlangen University Clinic and Würzburg University Clinic. The patients underwent surgical treatment in one of the university centers between 1995 and 2015. Epidemiological data, as well as tumor-related information and follow-up data, were collected prospectively. Common follow-up strategies were applied with regular physical examinations, estimation of carcinoembryogenic (CEA) levels, abdominoperineal ultrasonographies, and colonoscopies within the first 5 years after treatment. Patients with rectal cancer were additionally followed up with a computed tomography of the pelvis, rectoscopies, and chest X-rays. Subsequently, we identified the vital status by population registry or the general practitioner. The mean follow-up time was 84 months in UC and 52.2 months in CC. We lost 2 patients during follow-up, who did not take the follow-up examinations and had a mean observation time of 1.5 months. The follow-up documentation ended in October 2019. The following inclusion criteria were applied: presence of UC or CC; invasive adenocarcinoma, including signet ring cell carcinoma, mucinous adenocarcinoma, adenosquamous, or undifferentiated carcinoma; and localization of the carcinoma in the rectum or colon. The rectum is defined as being less than 16 cm from the anocutaneal line when measured with a rigid rectoscope. We excluded patients with neuroendocrine and squamous cell carcinoma or sporadic, non-inflammatory carcinogenesis, if confirmed by the pathologist. Cancers were detected either because of symptoms or during routine colonoscopies or as incidental findings in the context of therapeutic colectomies in UC patients. This patient collective of 88 patients, 63 from Erlangen and 25 from Würzburg, was divided into two groups according to the type of IBD: 20 patients with Crohn’s colitis and 68 with ulcerative colitis. These two groups were compared based on the following parameters: age at tumor diagnosis, disease duration, gender, tumor classification and localization of the tumor, surgical and therapeutic interventions, and presence of primary sclerosing cholangitis and survival. Tumors were examined and classified in accordance with the 4th edition of WHO Classification [12] and the 8th edition of TNM classification [13]. Rectal carcinomas were operated using total mesorectal excision (TME) and colon carcinomas with complete mesocolic excision (CME). According to the local ethics committee of the University of Erlangen and Würzburg, written consent was not necessary for this retrospective analysis. All patient-related data were analyzed anonymously.

The statistical software package SPSS® (version 21.0, IBM, Armonk, New York, USA) was used for data analysis. Continuous data, such as age, were presented with median value and the unpaired *t* test was used to compare the differences between the two groups. Categorical variables, e.g., tumor classification, were shown as relative and absolute frequencies and comparisons were performed using the Chi-square test or Fisher’s exact test. An effect was considered statistically significant at *p* < 0.05. The overall and the tumor-specific survival rates, as well as the disease-free survival rate, were calculated using the Kaplan-Meier method. In overall survival rate, death of any cause was considered an event. To compute the tumor-specific survival rate, only patients dying of CAC were used for calculation and every patient who was still alive, died of another cause than CAC, or was lost during follow-up, has been censored. In the disease-free survival rate, local recurrence, metachronous metastases, or death of different cause than CAC after initial R0 resection were taken as an event. The log-rank test was used to compare the survival curves among the groups. A multivariate Cox regression analysis of possible predictive factors was performed using significant parameters. For identification of independent prognostic factors, a univariate analysis was performed and all variables with *p* < 0.05 were included into the multivariate model.

## Results

### Patient characteristics and clinical features

We identified 88 patients with CAC (68 with ulcerative colitis and 20 with Crohn’s colitis), 51 (58%) men and 37 (42%) women. The median age was 26 (5–71) years at the onset of UC and 21 (14–48) years at the onset of CC. Median disease duration of the inflammatory bowel diseases until the detection of cancer was found to be similar with 19 (1–41) years in UC and 18 (4–32) years in CC, respectively (*p* = 0.840). The median age at tumor diagnosis was 49.5 (22–83) years in UC and 45.5 (28–65) years in CC (*p* = 0.208), which is both far below the reported average age in sporadic carcinoma (72.9 years in women and 70.3 years in men in Germany) [14]. Thirty-two (46.7%) patients with UC were diagnosed with cancer during routine colonoscopy, whereas 14 (70.6%) patients with CC showed symptoms like weight loss or intestinal obstruction, which led to the cancer detection (*p* = 0.013). In each group, approximately 10% also suffered from primary sclerosing cholangitis (PSC), a well-known risk factor for the development of CAC in patients with concomitant IBD [2]. Only one patient of those suffering from PSC underwent liver transplantation before diagnosis of CAC because of progressive liver cirrhosis. Twelve (18.2%) patients with UC and 5 (26.3%) patients with CC were controlled with immunosuppressive medication, in particular cyclosporine, azathioprine, methotrexate, or biologicals before the diagnosis of cancer (*p* = 0.517).

### Pathological features

Interestingly, 45.0% of the patients with CC (9 patients) compared with only 10.7% with UC (7 patients) had a signet ring cell (grading G3) or a mucinous type (grading G3) of adenocarcinoma, which is associated with a poorer survival prognosis (*p* = 0.002). Furthermore, there was a significant difference in comparing tumor localization in patients with UC and CC (*p* = 0.005). 66.2% of the patients with UC (45 patients) were diagnosed with colonic cancer, while in CC, 70.0% (14 patients) suffered from rectal cancer. We observed 8 (11%) patients in UC and 2 (10%) in CC with synchronous malignancies in the large bowel with up to 5 synchronous lesions in the patients with pancolitis ulcerosa. In 6 patients with CC, we found a fistula carcinoma. Corresponding to the tumor localization and recommendations of guidelines, 57.4% of the patients with UC had a proctocolectomy or a colectomy, while 35.0% with CC were treated with an abdominoperineal excision or a low anterior resection. We also found significant differences in the two groups concerning tumor invasion stratified in TNM classification. While 13 (19.1%) patients with UC were diagnosed with pT1 colorectal cancer, none of the patients with CC had this relatively early category. In contrast, we identified 8 patients (47.0%) in the CC group suffering from far advanced cancer in category pT4/ypT4 with tumor infiltration in neighboring organs or the peritoneum, compared with 14 patients (25%) with UC having the same condition (*p* = 0.008). Regarding the lymph node status, we were able to observe 16 (23.8%) patients with UC and 4 (23.5%) with CC having extensive lymph node infestation in category pN2 or ypN2 (*p* = 0.163). Fifteen (22.4%) patients in UC and 6 (30.0%) in CC were diagnosed with distant metastases and no difference could be seen in the two groups (*p* = 0.490). When stratified according to UICC stage, we were able to observe a trend towards more diagnoses with the highest stage, UICC IV, in CC in comparison with UC, although the difference did not reach a level of significance (*p* = 0.217). The postoperative residual of tumor tissue is described by the R classification. The distribution differed significantly between UC and CC patients (*p* = 0.046): 36.8% (7 patients) in CC versus 11.3% (7 patients) in UC were diagnosed with postoperative macroscopic residual (R2), e.g., local remains of macroscopic tumor tissue (2 patients) or non-operable distant metastases (12 patients). Those could be found in the liver (4 patients), the peritoneum (5 patients), or in multiple organs, including bone metastases (3 patients). Intraoperative radiation therapy for treatment of residual tumor was not applied to any of the patients. Nine patients (32.1%) out of all patients with rectal tumor in UICC stage II, III, or IV underwent neo-adjuvant therapy, which was either a short-term radiotherapy or a radiochemotherapy. Adjuvant radiotherapy was offered to 5 patients (6%) and adjuvant chemotherapy to 38 patients (45.3%), among these 66% with a curative approach and 34% palliative. Outcome differs significantly among the patients with curative and palliative approach (*p* < 0.001). Table 1 shows a comparison of the clinico-pathological features in the UC and CC groups.

**Table 1:** Clinico-pathological features of 88 patients with ulcerative colitis (68) and Crohn’s colitis (20)

	Ulcerative colitis (%)	Crohn’s colitis (%)	*p*
Total	68	20	
Median age at tumor diagnosis (years)	49.5	45.5	0.208
Sex			
Male	41 (60.2)	10 (50.0)	0.412
Female	27 (39.1)	10 (50.0)	
Median disease duration before CAC^1^ (years)	19.0	18.0	0.840
Median age at IBD^2^ diagnosis (years)	26.0	21.0	0.248
Localization			
Rectum	23 (33.8)	14 (70.0)	0.005
Colon	45 (66.2)	6 (30.0)	
T-category			
pT1	13 (19.1)	0 (0.0)	0.008
pT2	5 (7.3)	3 (17.6)	
pT3	33 (48.5)	4 (23.5)	
pT4	11 (20.6)	8 (47.0)	
ypT3	3 (4.4)	2 (11.8)	
ypT4	3 (4.4)	0 (0.0)	
pTx	0	3	
N-category			
pN0	37 (55.2)	7 (41.2)	0.163
pN1	9 (13.4)	4 (23.5)	
pN2	15 (22.4)	3 (17.6)	
ypN0	1 (1.4)	2 (11.8)	
ypN1	4 (5.9)	0 (0.0)	
ypN2	1 (1.4)	1 (5.9)	
pNx	1	3	
M-category			
M0	52 (77.6)	14 (70.0)	0.490
M1	15 (22.4)	6 (30.0)	
Mx	1	0	
Stage (UICC)			
Stage I	17 (25.0)	2(12.5)	0.217
Stage II	20 (29.4)	3 (18.8)	
Stage III	14 (20.6)	3 (18.8)	
Stage IV	11 (16.2)	6 (37.5)	
Stage yII	1 (1.5)	1 (6.2)	
Stage yIII	1 (1.5)	1 (6.2)	
Stage yIV	4 (5.9)	0 (0.0)	
Missing	0	4	
R classification			
R0	53 (85.5)	12 (63.2)	0.046
R1	2 (3.2)	0 (0.0)	
R2	7 (11.3)	7 (36.8)	
Rx	6	1	
Histological type			
Adenocarcinoma	56 (86.1)	11 (55.0)	0.002
Mucinous adenocarcinoma	7 (10.7)	6 (30.0)	
Signet ring cell carcinoma	0 (0.0)	3 (15.0)	
Adenosquamous carcinoma	1 (1.5)	0 (0.0)	
Undifferentiated carcinoma	1 (1.5)	0 (0.0)	
Missing	3	0	
Synchronous malignancies			
No	59 (86.7)	18 (90.0)	1
Yes, in the colon	8 (11.7)	2 (10.0)	
Yes, in other organs	1 (1.5)	0 (0.0)	
PSC^3^			
Yes	8 (12.3)	2 (10.5)	1
Operation methods			
Right hemicolectomy	7 (10.2)	5 (25.0)	0.007
Left hemicolectomy	7 (10.2)	4 (20.0)	
Sigmoid resection	2 (2.9)	0 (0.0)	
Low anterior resection	6 (8.8)	1 (5.0)	
Abdominoperineal excision	6 (8.8)	6 (30.0)	
Colectomy	14 (20.6)	2 (10.0)	
Proctocolectomy	25 (36.8)	1 (5.0)	
Non-radical resection	1 (1.5)	1 (5.0)	

^123^Colitis-associated cancer. Inflammatory bowel disease. Primary sclerosing cholangitis

### Survival analysis

**Fig. 1:**
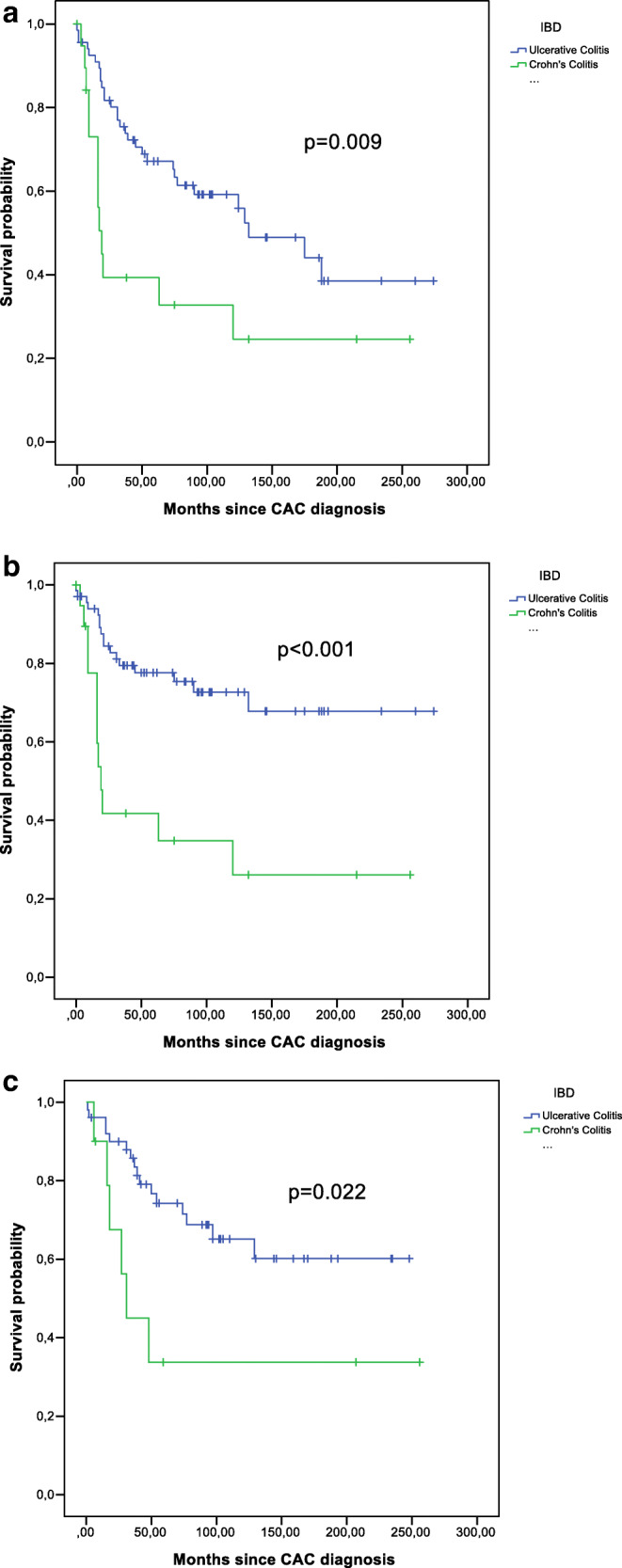
**a** Survival analysis for overall survival, **b** survival analysis for tumor-specific survival, and **c** survival analysis for disease-free survival

**Fig. 2:**
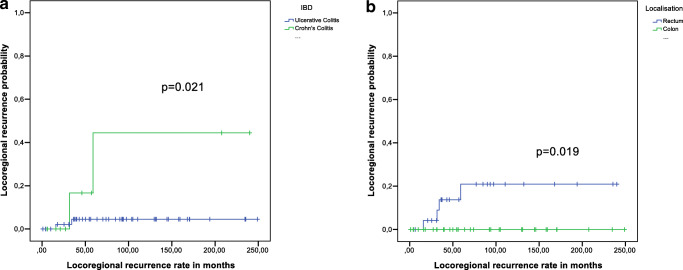
**a** Locoregional recurrence analysis for ulcerative colitis and Crohn’s colitis and **b** locoregional recurrence analysis for rectum and colon

A Kaplan-Meier survival curve showing overall survival by type of IBD and tumor-specific survival is presented in Fig. 1a, b. There was a significant difference in the comparison of overall survival between patients with CC and UC (*p* = 0.009).The 5-year survival rate after cancer in patients with UC was 67.1% and in patients with CC 39.3% (Table 2). The tumor-specific survival rate showed an even more significant divergence between CC and UC (*p* < 0.001). Disease-free survival rate proved to be significant as well (*p* = 0.022) (Fig. 1c). Regarding only the rate of locoregional recurrences, there was a significant difference in the two groups, with a higher rate in CC (*p* = 0.021) (Fig. 2a). In addition, when comparing tumors in rectum and colon, we observed that all locoregional recurrences occurred in patients with rectal tumors (*p* = 0.019) (Fig. 2b). Short-term survival rate after 30 days was 97.1% in UC and 100% in CC.

**Table 2:** 5-year survival rates

	*n*	5 YSR^1^	95% CI	*p*
Sex				
Male	51	60.7%	46.8–74.6%	0.900
Female	37	61.7%	45.2–87.2%	
Localization				
Rectum	37	61.9%	45.4–78.4%	0.952
Colon	51	60.3%	46.3–74.3%	
T-category				0.006
pT1	13	82.5%	60.4–100.0%	
pT2	8	66.7%	29.1–100.0%	
pT3	34	72.0%	57.3–86.7%	
pT4	19	31.6%	10.6–52.6%	
ypT3	5	00.0%		
ypT4	3	50.0%	0.0–100.0%	
N-category				
pN0	44	75.2%	61.9–88.5%	0.006
pN1	13	61.5%	35.0–88.0%	
pN2	18	43.2%	19.9–66.5%	
ypN0	3	66.7%	13.4–100.0%	
ypN1	4	37.5%	0.0–93.5%	
ypN2	2	00.0%		
M-category				
cM0	66	75.5%	64.7–86.3%	< 0.001
cM1	21	9.1%	0.0–24.6%	
Stage (UICC)				
Stage I	19	80.4%	60.4–100.0%	< 0.001
Stage II	23	77.4%	60.0–94.8%	
Stage III	17	88.2%	72.9–100.0%	
Stage IV	17	0.00%		
Stage yII	2	50%	0.0–100.0%	
Stage yIII	2	50%	0.0–100.0%	
Stage yIV	4	0.00%		
R classification				
R0	65	76.9%	66.3–87.5%	< 0.001
R1	2	00.0%		
R2	14	00.0%		
Histological type				
Adenocarcinoma	67	67.7%	64.9–88.5%	0.037
Non-adenocarcinoma	18	35.4%	12.7–58.1%	
IBD^2^				
Ulcerative colitis	68	67.1%	55.5–78.7%	0.009
Crohn’s colitis	20	39.3%	16.8–61.8%	
PSC^3^				
No	74	60.3%	48.6–72.0%	0.922
Yes	10	70.0%	41.6–98.4%	
Neoadjuvant therapy				
No	79	63.2%	52.0–74.4%	0.433
Yes	9	41.7%	7.8–75.6%	
Adjuvant therapy				
No	50	74.1%	60.8–87.4%	< 0.001
Yes, curative	25	74.6%	57.0–92.2%	
Yes, palliative	13	7.7%	0.0–22.2%	
Immunosuppressive medication				
No	68	60.7%	48.5–72.9%	0.637
Yes	17	62.5%	38.8–86.2%	

^123^5-year survival rate. Inflammatory bowel disease. Primary sclerosing cholangitis

### Cox regression analysis

In the univariate analysis, UICC disease stage IV was found to be a significant prognostic factor for overall survival (*p* < 0.001). In addition to UICC disease stage, factors associated with a poorer overall survival in univariate analysis were tumor stage pT4 (*p* = 0.003), lymph node metastases category pN2/ypN2 (*p* = 0.011/0.011), distant metastases (*p* < 0.001), postoperative microscopic (R1) or macroscopic (R2) residual tumor (*p* = 0.009/< 0.001), a non-adenocarcinoma histological type (*p* = 0.042), and Crohn’s colitis (*p* = 0.011) (Table 3). A multivariate Cox regression revealed a higher UICC tumor stage to be independently associated with a decreased overall survival after correction for histological type, postoperative residual, and IBD (*p* = 0.002). The difference regarding survival between UC and CC was no longer significant (*p* = 0.061).

**Table 3:** Cox regression

			Univariate analysis			Multivariate analysis		
		*n*	HR^1^	95% CI	*p*	HR^1^	95% CI	p
Sex	Male	51	1.0					
	Female	37	1.0	0.6–1.9	0.901			
Localization	Rectum	37	1.0					
	Colon	51	1.0	0.5–1.8	0.953			
T-category	pT1	13	1.0					
	pT2	8	2.6	0.4–18.7	0.336			
	pT3	37	4.0	0.9–17.0	0.064			
	pT4	19	9.4	2.1–41.4	0.003			
	ypT3	5	8.0	1.3–48.6	0.024			
	ypT4	3	5.5	0.5–60.8	0.166			
N-category	pN0	44	1.0					
	pN1	13	2.3	1.0–5.4	0.044			
	pN2	18	2.6	1.3–5.8	0.011			
	ypN0	3	0.9	0.1–6.6	0.890			
	ypN1	4	2.3	0.5–10.5	0.262			
	ypN2	2	18.4	1.9–172.9	0.011			
M-category	cM0	66	1.0					
	cM1	21	7.7	3.7–15.7	< 0.001			
Inflammatory bowel disease	Ulcerative colitis	68	1.0			1.0		
	Crohn’s colitis	20	2.3	1.2–4.5	0.011	2.3	1.0–5.6	0.061
Stage (UICC)	Stage I	19				1.0		
	Stage II	23	2.4	0.6–8.7	0.200	1.8	0.5–7.0	0.407
	Stage III	17	2.8	0.7–10.3	0.124	2.4	0.6–9.1	0.216
	Stage IV	17	39.7	9.7–161.2	< 0.001	22.3	3.3–151.6	0.002
	Stage yII	2	5.1	0.5–49.8	0.160	5.5	0.6–54.2	0.145
	Stage yIII	2	9.3	0.9–92.7	0.057	9.4	0.9–94.7	0.057
	Stage yIV	4	11.4	1.8–72.4	0.010	5.7	0.3–88.6	0.216
R classification	R0	65	1.0			1.0		
	R1	2	18.0	2.1–156.1	0.009	14.4	0.5–385.7	0.113
	R2	14	14.2	6.1–32.9	< 0.001	2.3	0.6–9.4	0.241
Histological type	Adenocarcinoma	67	1.0			1.0		
	Non-adenocarcinoma	18	2.0	1.0–4.0	0.042	1.2	0.5–3.0	0.681

^1^Hazard ratio

## Discussion

In this retrospective study, we found a significantly poorer survival for patients with cancer complicating CC in comparison with UC. In addition, patients with CC were found to have more advanced tumor stages and more aggressive histological types of cancer.

In our study, based on data documentation over a time span of 20 years, we identified 68 patients with UC and cancer, but only 20 patients with CC and cancer. This might be due to a lower risk of colorectal cancer in CC (SIR 2.4 in UC, but 1.9 in CC). However, another possible explanation could be the lower incidence of CC in the general population (322 per 100.000 persons) compared with UC (505 per 100.000 persons) [15].

Long disease duration and early age at disease onset of IBD [16] are independent risk factors for developing CAC [17, 18]. Averboukh et al. reported a mean disease duration of 22.7 years in UC and 16.6 years in CC, before diagnosis of colorectal cancer. Svrceck et al. found a median disease duration of 20.7 years in UC and 15.4 years in CC. Our findings of a median disease duration of 19.0 years in UC and 18.0 years in CC are comparable with the published data, confirming a long disease duration as a risk factor of cancer development. Interestingly, there was a significant difference in the circumstances of cancer detection between UC and CC: 70.6% of the patients with CC were diagnosed because of symptoms and not during routine colonoscopies, as in the case of UC patients. This may indicate a lower rate of surveillance of CC patients by colonoscopies compared with UC patients.

Immunomodulators (thiopurines or methotrexate) and biologic agents, such as TNF-α inhibitors, are commonly used in the treatment of IBD, but are suspected to promote carcinogenesis by direct genomic alteration or inhibition of physiologic immunosurveillance [19]. Pasternak et al. showed an increased risk of overall cancer in patients with IBD using azathioprine with a rate ratio of 1.41 [20]. In our study, UICC stage or survival rate after diagnosis of CAC did not differ between the patients who were controlled with immunomodulators and those who were not (*p* = 0.637).

With respect to typical features of CAC, we observed a young age at tumor diagnosis, multiple lesions, and a frequent occurrence of mucinous adenocarcinoma and signet ring cell carcinoma [21, 22]. Interestingly, we found a significantly higher frequency of mucinous and signet ring cell tumors in our study in CC compared with UC whereas other studies show no difference between these two groups [23, 24]. These two histological types are known for a more aggressive tumor type and a poor prognosis and could therefore contribute to poor survival rates in patients with CC [25].

Concerning the anatomical distribution of tumors in UC, most authors describe an occurrence on the left-sided colon [23]. This coincides with the typical localization of inflammation in UC patients, arising in the rectum and expanding proximately. However, other studies have identified different localizations of cancer in CC. Choi et al. reported a high incidence of tumors in the right colon [23], whereas others described a predominance of cancer in the rectosigmoid region [24]. We observed a significant difference in the anatomical distribution of the tumors between CC and UC. CC patients had a high incidence of rectal cancers, which is in line with the findings in the aforementioned studies and can be explained by an active inflammation with fistulas in this region in the majority of the patients. In contrast to these findings, most of the UC patients were diagnosed with cancer in the right-sided colon. Taking into consideration that all patients with UC had cancer in inflamed areas, the expansion of inflammation to the right-sided colon or pancolitis respectively might be an explanation for the localization of cancer in our patient cohort.

A close look to current therapy guidelines of CAC may be helpful to explain the difference in local recurrence rates between UC and CC: in UC proctocolectomy or colectomy are the treatments of choice [26], whereas in patients with CC less extensive operation procedures are performed to preserve as much of the large bowel as possible. Our study showed a high local recurrence rate of 44.4% within 5 years in patients with CC (Fig. 2a). Additionally, we found a high rate of locoregional recurrences in patients with rectum carcinomas, which are more frequent in our CC patients. Therefore, a reconsideration of the current operation strategy towards a more rigorous surgical intervention needs to be discussed to reduce the amount of local relapses in CC patients.

Leowardi et al. reported more advanced tumor stages in patients with colorectal cancer complicating UC compared with sporadic colorectal carcinoma [21]. In contrast, Watanabe et al. did not find a significant difference in the distribution of tumor stage between UC and sporadic cancer [22]. Regarding tumor stage in CC, Kersting et al. described advanced tumor stages in all CC patients included in their study [27]. We observed a high amount of fairly advanced tumors in both groups. UICC tumor stage, comprising tumor depth, lymph node metastases, and distant metastases, tends to more often be advanced in patients with CC compared with those with UC (37.5% UICC stage IV/yIV in CC versus 22.1% in UC, not significant). Looking at tumor depth, T-category was significantly higher in CC than in UC. A possible explanation could be a deeper expansion of inflammation as a patho-morphological feature of CC. Another reason for the advanced invasion could be a later diagnosis of cancer in CC, probably due to neglected surveillance in patients with CC.

There is no agreement in literature about survival prognosis after diagnosis of colorectal cancer in patients with UC compared with sporadic cancer. In a large population-based Danish study, Jensen et al. reported a poorer survival for patients with UC compared with sporadic cancer, while Leowardi et al. did not find a significant prognostic difference. In CC, Larsen et al. described an unfavorable prognosis in comparison with sporadic colorectal cancer with a hazard ratio of 1.82 after 1 year of follow-up [28]. We found a significant difference in the overall and tumor-specific survival comparing CC and UC (*p* = 0.009, *p* < 0.001), with a worse survival rate for CC. In multivariable Cox regression, a higher UICC tumor stage seemed to be a significant independent risk factor for overall survival with a 22.3-fold higher risk at UICC stage IV compared with stage I. After correction for tumor stage, the survival difference between UC and CC was no longer significant. Therefore, a higher tumor stage could be a possible explanation for the significantly lower survival in patients with CC. In some studies comparing both IBDs, adverse tumor prognosis and advanced stages were also described in patients with CC, compatible with our findings. Averboukh et al., for example, reported a significantly higher amount of patients with CC complicating cancer in an advanced stage, but the lower 5-year survival rate in CC could not reach a level of significance. Others could not find a significant difference between CC and UC regarding advanced tumor stages and survival prognosis [23].

Recent guidelines recommend a risk adapted colorectal cancer surveillance for patients with UC. High-risk patients with extensive colitis and severe inflammation or primary sclerosing cholangitis should have surveillance colonoscopy once a year, while patients with intermediate risk features, like colitis with moderate inflammation, should have their next surveillance scheduled in 2 to 3 years. A 5 year interval is recommended on colitis patients with neither high nor intermediate risk for colorectal cancer [29]. Data about the effectiveness of colonoscopic surveillance are still limited, but studies evaluating the consequences suggest a positive impact [30]. While having already established a surveillance program for patients with UC for decades, the first published data describing a surveillance program for CC patients is published in 2001 by Friedman et al. [31, 32]. Less attention is given to the screening colonoscopy for patients with Crohn’s Colitis, due to the long time unknown–elevated cancer risk in these patients [33]. It was only in recent times that the same standards of colonoscopic surveillance are applied for patients with CC as for UC patients [34]. Therefore, a possible explanation for the poor prognosis and the advanced tumor stages in our patients with colorectal cancer complicating CC could be attributed to less colonoscopic surveillance. The risk of colorectal cancer in patients with CC should not be underestimated, and medical practitioners and patients should be aware of this risk and the need of surveillance. As a consequence, specific surveillance advices and therapeutic methods tailored to patients with CC should be integrated in the guidelines.

In summary, we studied clinico-pathological features and survival rates of patients with colitis-associated cancer of UC and CC cases, until now being insufficiently compared. The retrospective design of our study and the origin of the data from two different medical centers might lead to a selective treatment bias, and we are aware that an unequal amount of cases of CC in comparison with UC might influence the findings in our study as well. Even though we conducted a small study, the significance of our results show that further research in the field of colitis-associated cancer needs to be performed to improve the prognosis of both patient groups.
